# A retrospective study on Xpert MTB/RIF for detection of tuberculosis in a teaching hospital in China

**DOI:** 10.1186/s12879-020-05004-8

**Published:** 2020-05-24

**Authors:** Shuguang Li, Liyan Lin, Feifei Zhang, Chunjiang Zhao, Han Meng, Hui Wang

**Affiliations:** Department of Clinical Laboratory, Peking University People’s Hospital, Xizhimen South Avenue No.11, Beijing, 100044 China

**Keywords:** Xpert MTB/RIF, Tuberculosis, Pulmonary tuberculosis, Extra-pulmonary tuberculosis, Tuberculous pleurisy

## Abstract

**Background:**

The Xpert MTB/RIF assay is an automated molecular test that is designed to simultaneously detect *Mycobacterium tuberculosis* (MTB) complex and rifampin resistance. However, there are relatively few studies on this method in China. Xpert has been routinely used at Peking University People’s Hospital (PKUPH) since November 2016. Thus, the aim of this study was to evaluate the performance of Xpert, and provide a reference and guidance for the detection and diagnosis of TB in non-TB specialized hospitals.

**Methods:**

The medical records of inpatients simultaneously tested with Xpert, acid-fast bacilli (AFB) smear microscopy, and interferon-gamma release assay (IGRA, by T-SPOT®.TB) at PKUPH from November 2016 to October 2018 were reviewed. Active TB cases were considered according to a composite reference standard (CRS). Then, the three methods were evaluated and compared.

**Results:**

In total, 787 patients simultaneously tested with Xpert, AFB, and IGRA were enrolled; among them 11.3% (89/787) were diagnosed and confirmed active pulmonary TB (PTB, 52 cases), extrapulmonary TB (EPTB, 17 cases), and tuberculous pleurisy (TP, 20 cases). The sensitivity of Xpert in detecting PTB, EPTB, and TP was 88.5, 76.5, and 15.0%, respectively, which was slightly lower than IGRA (96.2, 82.4, and 95.0%, respectively), but higher than AFB (36.5, 11.8, and 0%, respectively); IGRA showed the highest sensitivity, but its specificity (55.9, 67.1, and 45.2%, respectively) was significantly lower than Xpert (99.6, 99.4, and 100%, respectively) and AFB (99.0, 99.4, and 100%, respectively) (*P* < 0.001). The sensitivity of Xpert in detecting lung tissue, cerebrospinal fluid, lymph nodes, and joint fluid was 100%, followed by sputum (88.5%), alveolar lavage (85.7%), and bronchoscopy secretion (81.2%); the pleural fluid sensitivity was the lowest, only 15.0%. For AFB negative patients, the sensitivity of Xpert in detecting PTB, EPTB, and TP was 84.9, 73.3, and 15.0%, respectively.

**Conclusions:**

Xpert showed both high sensitivity and high specificity, and suggested its high value in TB diagnosis; however, the application of pleural fluid is still limited, and should be improved. Owing to the high sensitivity of IGRA, it is recommended for use as a supplementary test, especially for assisting in the diagnosis of TP and EPTB.

## Background

Tuberculosis (TB), an infectious disease caused by *Mycobacterium tuberculosis* (MTB) complex, usually affects the lungs but also affects other parts of the body [1]. The typical symptoms of active pulmonary TB (PTB) are chronic cough and hemoptysis, fever, night sweats, and weight loss. Currently, about a quarter of the world’s population are carriers of MTB [2]. In 2018, there were an estimated 10.0 million new TB cases globally; of these, China accounted for 9% of the global total [2]. There were 1.3 million deaths from TB in 2017, and TB became the leading cause of infectious diseases all over the world [2, 3]. Notably, extrapulmonary TB (EPTB) is also emerging as a serious clinical problem, and comprises an increased proportion of total TB cases in the past few decades [4–6].

Traditionally, the diagnosis of active TB is mainly based on chest X-ray, microscopy, and body fluid culture; whilst the diagnosis of latent TB depends on the tuberculin skin test or hematology test. Histology and X-ray relies on highly trained operators, and characteristic morphology is shared with other diseases. Acid-fast bacilli (AFB) smear microscopy remains the most used and widely available TB diagnostic method in low-income and middle-income countries; however, as many as 40–50% of active TB cases were smear-negative [7]. TB culture requires 2–6 weeks for interpretation [2], and has less than perfect sensitivity [8, 9]; thus culture was not done with all presumptive patients in non-TB specialized hospitals in China. Interferon-gamma (IFN-γ) release assay (IGRA) is a new immunoassay for TB diagnosis, and has been widely applied throughout China in recent years; however, the heterogeneity of diagnostic efficacy in active TB samples varies from 50 to 100%, with a specificity of 83–98% [10, 11]. Thus, the current diagnosis of TB is still challenging.

The rapid test Xpert MTB/RIF (Cepheid, Sunnyvale, CA, USA), an automated real-time PCR platform that can detect both MTB complex and rifampicin resistance within two hours, has been recommended by the World Health Organization (WHO) as the initial diagnostic test in all persons with signs and symptoms of TB [12]. Xpert MTB/RIF has been well documented in the literature in many countries worldwide; however, as a country with large numbers of TB patients, there are relatively few studies on this method in China. Xpert MTB/RIF has been routinely used in Peking University People’s Hospital (PKUPH), a comprehensive teaching hospital in Beijing, China, since November 2016. Thus, the aim of this study was to evaluate the performance of Xpert MTB/RIF, and provide a certain reference and guidance for the detection and diagnosis of TB in non-TB specialized hospitals.

## Methods

### Study design

This is a retrospective survey and analysis of data collected for clinical purposes. Inpatients simultaneously tested with Xpert MTB/RIF, AFB smear microscopy, and IGRA at PKUPH from November 2016 to October 2018 were included. PKUPH is a non-TB specialized, comprehensive teaching hospital, which serves patients in Beijing, as well as surrounding cities in northern China, and even throughout China. Equipped with over 2000 beds, the hospital admits more than 78,000 inpatients and handles nearly 2.6 million outpatient visits each year.

### Acid-fast bacilli (AFB) smear microscopy

Smear microscopy was performed according to the Clinical and Laboratory Standards Institute (CLSI) M48-A guideline [13]. Specimens were stained for acid-fast microscopic examination using the Ziehl-Neelsen stain (BaSO Diagnostics Inc., Zhuhai, China). Smear-positive specimens were graded from 1+ to 4+ according to the American Thoracic Society scale [14].

### Interferon-gamma release assay (IGRA)

The heparinized blood of patients was collected and used for the T-SPOT®.TB test (Oxford Immunotec, Oxford, UK) in accordance with the manufacturer’s instructions. When the negative and positive quality controls were under control, the results were considered positive if either panel A or panel B had ≥6 spots number.

### Xpert MTB/RIF assay

Xpert MTB/RIF assay was performed on the GeneXpert Dx instrument system according to the manufacturer’s recommendations (Cepheid, Sunnyvale, CA, USA) (Detailed in supplementary material 1). The Xpert software was used to interpret the results, and semiquantitative results were provided based on the cycle threshold (CT) defined by the manufacturer as follows: high (CT ≤ 16), medium (16 < CT ≤ 22), low (22 < CT ≤ 28), and very low (CT > 28).

### Classification and diagnosis

Active TB cases were considered comprehensively according to a composite reference standard (CRS), which combined the WHO guidelines [15] and WS 288--2017 (released on 2017-11-09 and implemented on 2018-05-01) by the National Health and Family Planning Commission of the People’s Republic of China (NHFPC, is now National Health Commission of the People’s Republic of China) [16]. Briefly, its contents are as follows: (1) microbiologically confirmed PTB: patients with positive MTB culture, or a pulmonary case with one or more positive initial sputum smears and chest imaging; (2) molecular biologically confirmed PTB: patients with positive MTB nucleic acid test and chest imaging; (3) histopathological examination confirmed PTB: patients with positive histopathological examination; (4) EPTB: patients with definite TB involving organs other than the lungs, with MTB isolated from a non-pulmonary source or histological or strong clinical evidence consistent with active EPTB, as well as improvement observed in anti-TB specific therapy. Thus, CRS was a combination of many pertinent aspects, which could diagnose the culture-negative TB and provide a higher accuracy. The cases were categorized as confirmed TB (PTB, EPTB, or tuberculous pleurisy, i.e., TP) or non-TB; the concurrent PTB and EPTB/TP cases were classified as PTB [15]. Then, different methods were evaluated and compared.

## Results

### Patient characteristics

**Fig. 1:**
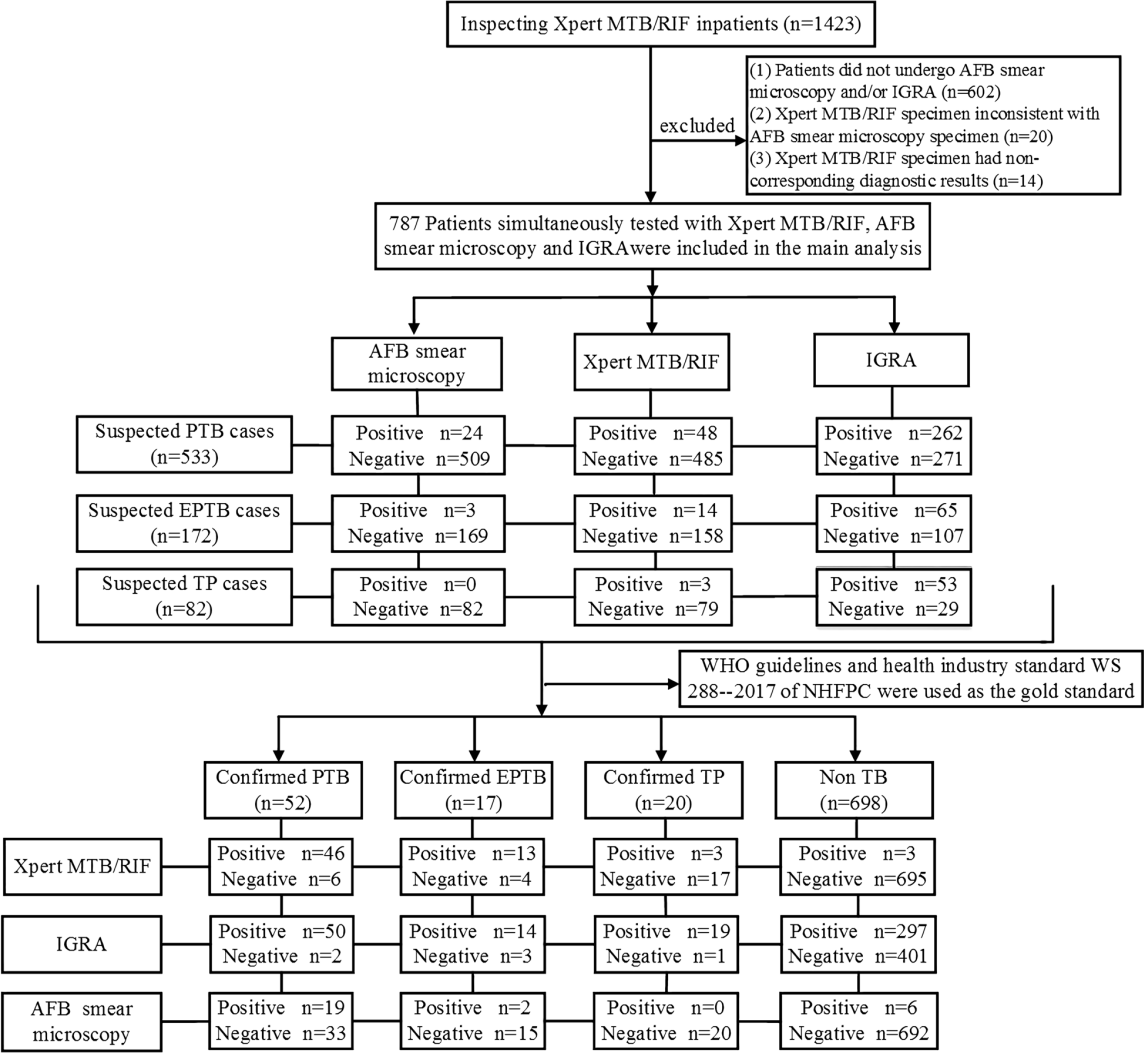
Flowchart explaining the overall patient flow and diagnostic classifications. AFB, Acid-fast bacilli; Interferon-gamma release assay, IGRA; TB, tuberculosis; PTB, pulmonary tuberculosis; EPTB, Extrapulmonary tuberculosis; TP, tuberculous pleurisy

From November 2016 to October 2018, 1423 patients were tested using the Xpert in PKUPH. Patients who did not undergo AFB smear microscopy and/or IGRA (*n* = 602), patients whose Xpert specimen was inconsistent with AFB smear microscopy (*n* = 20) or had non-corresponding diagnostic results (*n* = 14) were excluded. In all, 787 patients were enrolled in this survey (Fig. 1). The mean age of the patients was 55.4 ± 18.5 years, and 55.1% of them were males (Table S1). Combined with presumptive symptoms, specimens, and clinical diagnosis, the patients were divided into three categories: presumptive PTB (533 cases), presumptive EPTB (172 cases) and presumptive TP (82 cases) (Table 2). When using CRS as the gold standard, 89 patients (11.3%) were confirmed as active TB: 52 PTB, 17 EPTB, and 20 TP (Fig. 1).

### Comparison of Xpert MTB/RIF, AFB smear microscopy, and IGRA

**Fig. 2:**
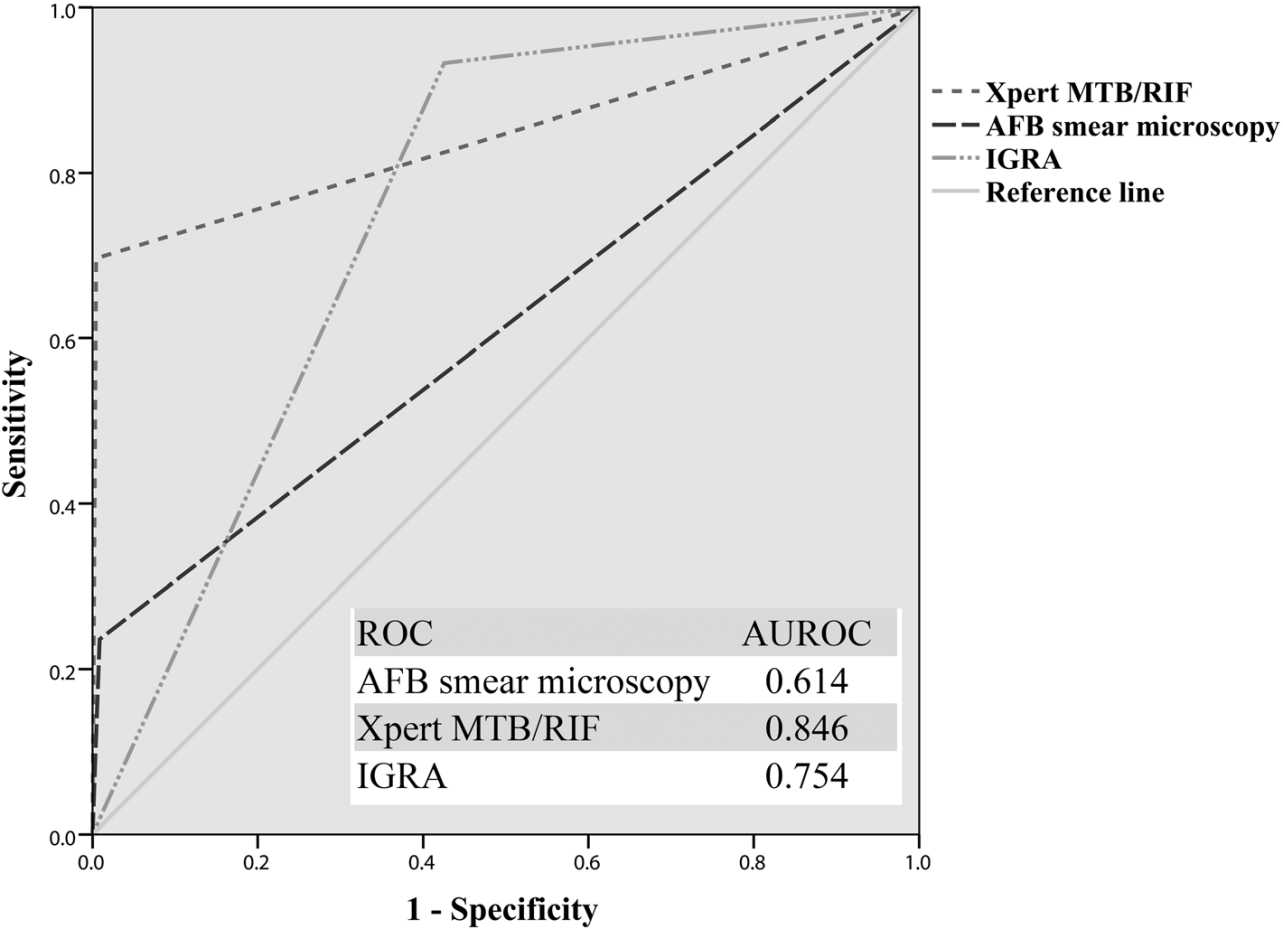
Receiver operating curves (ROC) for Xpert MTB/RIF, Acid-fast bacilli (AFB) smear microscopy and Interferon-gamma release assay (IGRA) to differentiate tuberculosis (TB) infection and non-TB infection cases. AUROC, area under the ROC

The positive ratios of AFB, Xpert, and IGRA were 3.4% (27/787), 8.3% (65/787), and 48.3% (380/787), respectively. Figure 2 shows the received operating characteristic (ROC) curves with area under the curve (AUC) for Xpert, AFB smear microscopy, and IGRA in comparison to the gold standard. The diagnostic performance of IGRA (AUC = 0.754, 95% confidence interval, i.e., 95% CI 0.722–0.783; sensitivity = 93.3, 95% CI 86.1–96.9%; specificity = 53.5, 95% CI 53.8–61.1%) was significantly higher than AFB (AUC = 0.614, 95% CI 0.579–0.648; sensitivity = 23.6, 95% CI 16.0–33.4%; specificity = 99.1, 95% CI 98.1–99.6%) (*P* < 0.001, Z test). Xpert showed the best diagnostic performance, with AUC value 0.846 (95% CI 0.819–0.871), sensitivity 69.7% (95% CI 59.5–78.2%), specificity 99.6% (95% CI 98.7–99.9%), and significantly higher than the other two methods (*P* < 0.001) (Fig. 2 and Table 1).

**Table 1:** Performance of Xpert MTB/RIF, AFB smear microscopy and IGRA in detecting PTB, EPTB and TP

	Sensitivity (95% CI) (%)	Specificity (95% CI) (%)	Positive predictive value (95% CI) (%)	Negative predictive value (95% CI) (%)
**Presumptive PTB cases (*n*** **= 533)**				
Xpert MTB/RIF	88.5 (77.0–94.6)	99.6 (98.5–99.9)	95.8 (86.0–98.9)	98.8 (97.3–99.4)
AFB smear microscopy	36.5 (24.8–50.1)^b^	99.0 (97.6–99.9)	79.2 (59.5–90.8)	93.5 (91.0–95.4)
IGRA	96.2 (87.0–98.9)	55.9 (51.6–60.3) ^b^	19.1 (14.8–24.3)	99.3 (97.4–99.8)
**Presumptive EPTB cases (*n*** **= 172)**				
Xpert MTB/RIF	76.5 (52.7–90.4)	99.4 (96.4–99.9)	92.9 (68.5–98.7)	97.5 (93.7–99.0)
AFB smear microscopy	11.8 (3.3–34.3) ^b^	99.4 (96.4–99.9)	66.7 (20.8–93.9)	91.1 (85.9–94.6)
IGRA	82.4 (59.0–93.8)	67.1 (59.4–74.0) ^b^	21.5 (13.3–33.0)	97.2 (92.1–99.0)
**Presumptive TP cases (*n*** **= 82)**				
Xpert MTB/RIF	15.0 (5.2–36.0)	100.0 (94.2–100.0)	100.0 (43.9–100.0)	78.5 (68.2–86.1)
AFB smear microscopy	0.0 (0.0–16.1)	100.0 (94.2–100.0)	NA (NA) ^a^	75.6 (65.3–83.6)
IGRA	95.0 (76.4–99.1) ^b^	45.2 (33.4–57.5) ^b^	35.9 (24.3–49.3)	96.6 (82.8–99.4)
**Total (*n*** **= 787)**				
Xpert MTB/RIF	69.7 (59.5–78.2)	99.6 (98.7–99.9)	95.4 (87.3–98.4)	96.3 (94.6–97.4)
AFB smear microscopy	23.6 (16.0–33.4) ^b^	99.1 (98.1–99.6)	77.8 (59.2–89.4)	91.1 (88.8–92.9)
IGRA	93.3 (86.1–96.9) ^b^	57.5 (53.8–61.1) ^b^	21.8 (18.0–26.3)	98.5 (96.8–99.3)

Abbreviation: *AFB* acid-fast bacilli; *IGRA* interferon-gamma release assay; *PTB* pulmonary tuberculosis; *EPTB* extra-pulmonary tuberculosis; *TP* tuberculous pleurisy; *CI* confidence interval. ^a^, NA, No AFB smear microscopy positive results were obtained in presumptive TP cases, thus the positive predictive value was not available. ^b^, statistical differences between the sensitivity or specificity of AFB or IGRA and Xpert MTB/RIF, chi-square (χ^2^) test, *P* < 0.001

**Fig. 3:**
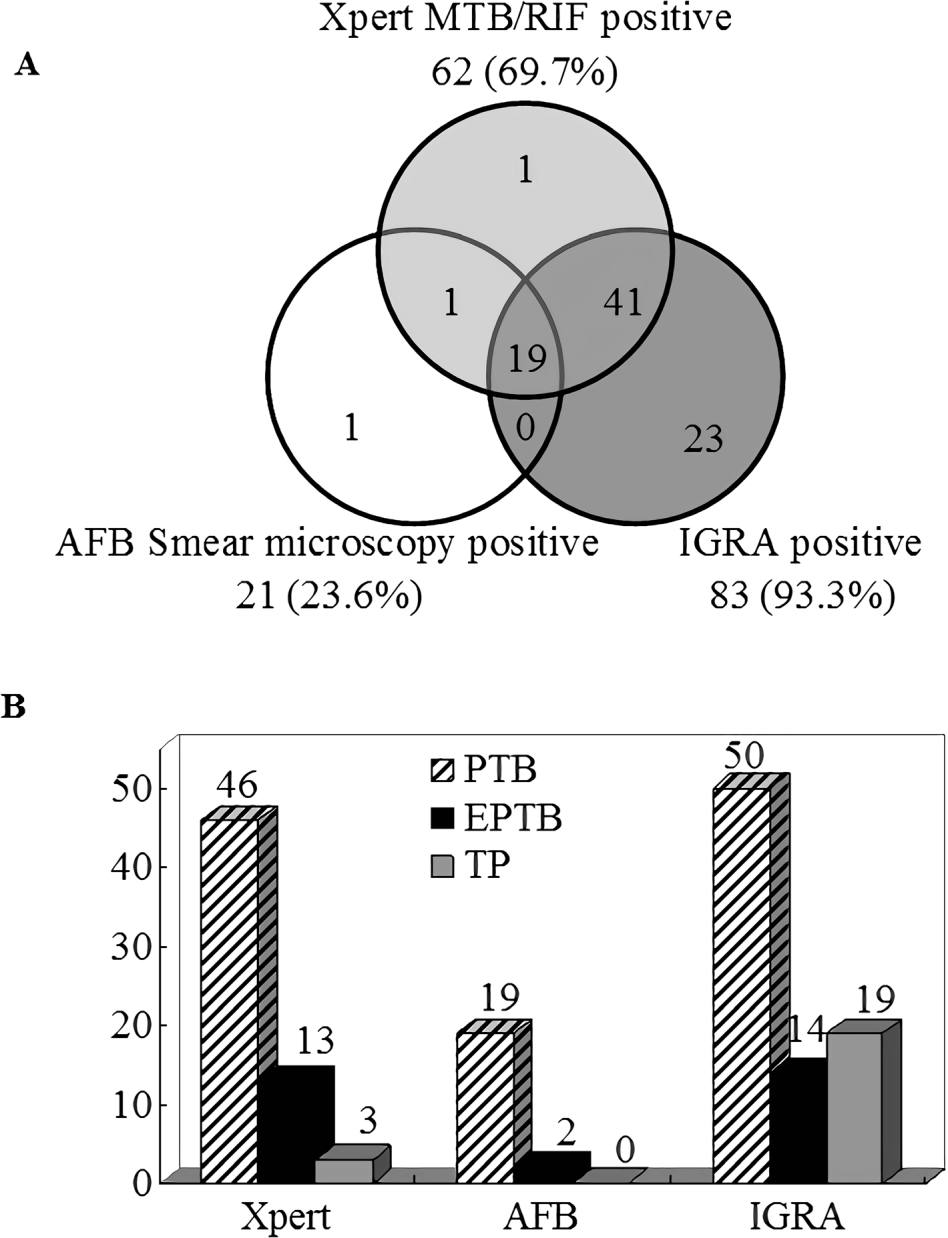
**a** Venn diagram of overlap in TB detection using Xpert MTB/RIF, Acid-fast bacilli (AFB) smear microscopy and Interferon-gamma release assay (IGRA); and **b** The number of true positive tuberculosis cases detected by the three methods. PTB, pulmonary tuberculosis; EPTB, Extrapulmonary tuberculosis; TP, tuberculous pleurisy

In presumptive PTB, EPTB, and TP cases, the positive ratios of Xpert were 9.0, 8.1, and 3.7%, respectively, which were higher than AFB (4.5, 1.7%, and 0), but lower than IGRA (49.2, 37.8, and 64.6%). Among the 89 confirmed TB cases, 19 cases (21.3%) tested positive using all the three methods, and the use of these three methods simultaneously could screen 96.6% (95% CI 90.5–98.9%) TB patients (Fig. 3a); 62 (69.7%, 46 PTB, 13 EPTB, and 9 TP), 21 (23.6%, 19 PTB, 2 EPTB, and 0 TP) and 83 (93.3%, 50 PTB, 14 EPTB, and 19 TP) cases tested positive using Xpert, AFB, and IGRA, respectively (Fig. 3b), while 3 (3.4%) confirmed TB cases (1 TP and 2 EPTB, i.e., 1 lumbar spine TB and 1 arthritis TB) tested negative using all the three methods. As shown in Table 1, the sensitivity of Xpert in detecting PTB, EPTB, and TP was 88.5% (95% CI 77.0–94.6%), 76.5% (95% CI 52.7–90.4%) and 15.0% (95% CI 5.2–36.0%), respectively, slightly lower than IGRA (96.2, 82.4, and 95.0%), but higher than AFB (36.5, 11.8, and 0%). IGRA had the highest sensitivity, but the specificity (55.9, 67.1, and 45.2%) was significantly lower than Xpert (99.6, 99.4, and 100%) and AFB (99.0, 99.4, and 100%) (*P* < 0.001); notably, the positive predictive values (PPV) (7.3, 95% CI 4.9–10.7%) is poor when only IGRA is positive.

### Performance of Xpert MTB/RIF assay in different specimens

As shown in Table 2, the highest sensitivity of Xpert in pulmonary specimens was in lung tissue (100%), followed by sputum (88.5%), BALF (85.7%), and FOB (81.2%); the specificity of all pulmonary specimens was higher than 99%. When detecting extrapulmonary specimens, the sensitivity and specificity of CSF, joint cavity fluid, and lymph node specimen were 100%; while the sensitivity for urine was relatively low (33, 95% CI 6.2–79.2%). The specificity of intestine tissue is 98.1%; other extrapulmonary specimens (except pleural fluid) showed a specificity of 100%. Notably, the sensitivity of pleural fluid was the lowest, only 15.0% (95% CI 5.2–36.0%), and significantly lower than pulmonary tissue, CSF, joint cavity fluid, extrapulmonary lymph node, sputum, BALF, and FOB (*P* < 0.05).

**Table 2:** Performance of Xpert MTB/RIF in different specimens

Specimen type	No. (%)	Xpert positive cases (%)	Confirmed TB (%)	Sensitivity (95% CI) (%)	Specificity (95% CI) (%)	Positive predictive value (95% CI) (%)	Negative predictive value (95% CI) (%)
**Presumptive PTB cases**	**533 (67.7%)**	**48 (9.0%)**	**52** (9.8%)	**88.5 (77.0–94.6)**	**99.6 (98.5–99.9)**	**95.8 (86.0–98.9)**	**98.8 (97.3–99.4)**
BALF	204 (25.9%)	7 (3.4%)	7 (3.4%)	85.7 (48.7–97.4)	99.5 (97.2–99.9)	85.7 (48.7–97.4)	99.5 (97.2–99.9)
Sputum	165 (21.0%)	24 (14.5%)	26 (15.8%)	88.5 (71.0–96.0)	99.3 (96.0–99.9)	95.8 (79.8–99.3)	97.9 (93.9–99.3)
FOB	105 (13.3%)	9 (8.6%)	11 (10.5%)	81.2 (52.3–94.9)	100.0 (96.1–100.0)	100.0 (70.1–100.0)	97.9 (92.7–99.4)
Lung tissue	52 (6.6%)	7 (13.5%)	7 (13.5%)	100.0 (64.6–100.0)	100.0 (92.1–100.0)	100.0 (64.6–100.0)	100.0 (92.1–100.0)
Others	7 (0.9%)	1 (14.3%)	1 (14.3%)	100.0 (20.7–100.0)	100.0 (61.0–100.0)	100.0 (20.7–100.0)	100.0 (61.0–100.0)
**Presumptive EPTB cases**	**172 (21.9%)**	**14 (8.1%)**	**17** (9.9%)	**76.5 (52.7–90.4)**	**99.4 (96.4–99.9)**	**92.9 (68.5–98.7)**	**97.5 (93.7–99.0)**
Intestine tissue	53 (6.7%)	1 (1.9%)	0 (0%)	NA^#^	98.1 (90.1–99.7)	0.0 (0.0–79.4)	100.0 (93.1–100.0)
CSF	42 (5.3%)	2 (4.8%)	2 (4.8%)	100.0 (34.2–100.0)	100.0 (91.2–100.0)	100.0 (34.2–100.0)	100.0 (91.2–100.0)
Ascitic fluid	18 (2.3%)	0 (0%)	0 (0%)	NA	100.0 (82.4–100.0)	NA	100.0 (82.4–100.0)
Joint cavity fluid	13 (1.7%)	2 (15.4%)	2 (15.4%)	100.0 (34.2–100.0)	100.0 (74.1–100.0)	100.0 (34.2–100.0)	100.0 (74.1–100.0)
Extrapulmonary lymph node	12 (1.5%)	5 (41.7%)	5 (41.7%)	100.0 (56.6–100.0)	100.0 (64.6–100.0)	100.0 (56.6–100.0)	100.0 (64.6–100.0)
Urine	11 (1.4%)	1 (9.1%)	3 (27.3%)	33.3 (6.2–79.2)	100.0 (67.6–100.0)	100.0 (20.7–100.0)	80.0 (49.0–94.3)
Others	23 (2.9%)	3 (13.0%)	5 (21.7%)	60.0 (23.1–88.2)	100.0 (82.4–100.0)	100.0 (43.9–100.0)	90.0 (69.9–97.2)
**Presumptive TP cases**	**82 (10.4%)**	**3** (3.7%)	**20** (24.4%)	**15.0 (5.2–36.0)**	**100.0 (94.2–100.0)**	**100.0 (43.9–100.0)**	**78.5 (68.2–86.1)**
Pleural fluid	82 (10.4%)	3 (3.7%)	20 (24.4%)	15.0 (5.2–36.0)	100.0 (94.2–100.0)	100.0 (43.9–100.0)	78.5 (68.2–86.1)
**Total**	**787**	**65** (8.3%)	**89** (11.3%)	**69.7 (59.5–78.2)**	**99.6 (98.7–99.9)**	**95.4 (87.3–98.4)**	**96.3 (94.6–97.4)**

Abbreviation: *PTB* pulmonary tuberculosis; *EPTB* extra-pulmonary tuberculosis; *TP* tuberculous pleurisy; *CI* confidence interval; *BALF* bronchoalveolar lavage fluid; *FOB* Fiberoptic bronchoscopy; *CSF* Cerebrospinal fluid. ^#^, NA, No Xpert positive results (and no confirmed TB cases) were obtained, thus the sensitivity (and positive predictive value) was not available

### Correlation between Xpert semiquantitative results and AFB smear microscopy results

The correlation between Xpert semiquantitative category and AFB smear grade is presented as cross-tabulated data (Table 3). Of 65 Xpert positive cases, 12 were positive high and 13 were positive medium, and PPV were all 100%; two and one false positive cases were obtained in positive low and positive very low cases, respectively. The sensitivity of AFB decreased gradually, accompanied with the Xpert results from 75.0% in Xpert positive high cases to 3.7% in Xpert negative cases. Notably, the PPV of AFB suddenly dropped from 100% in Xpert positive cases to 14.3% in Xpert negative cases, i.e., six of the seven (85.7%) positive AFB cases that occurred in Xpert negative cases were false positive. Totally, the PPV of Xpert (95.4, 95% CI 87.3–98.4%) were significantly higher than AFB (77.8, 95% CI 59.2–89.3%) (*P* < 0.05). Besides, the smear grades increased as the Xpert CT values decreased among the 62 Xpert true positive cases (Figure S1), and there was a strong, negative correlation between smear grades and Xpert CT values, which was statistically significant (R = -0.632, *P* < 0.001).

**Table 3:** Correlation between Xpert MTB/RIF semi-quantitative results and AFB smear microscopy results

	Prediction of Xpert positive				AFB Smear results					Prediction of AFB smear positivity		
		Total samples	Confirmed TB	PPV (95% CI)	Negative	1+	2+	3+	4+	Total smear positive	Sensitivity (95% CI)	PPV (95% CI)
Xpert results	Positive high	12	12	100% (75.8–100)	3	2	2	5	0	9	75.0% (46.8–91.1)	100% (70.1–100)
	Positive medium	13	13	100% (77.2–100)	6	6	0	1	0	7	53.9% (29.1–76.8)	100% (64.6–100)
	Positive low	23	21	91.3% (73.2–97.6)	21	2	0	0	0	2	9.5% (2.7–28.9)	100% (34.2–100)
	Positive very low	17	16	94.1% (73.0–99.0)	15	2	0	0	0	2	12.5% (3.5–36.0)	100% (34.2–100)
	Negative	722	27	NPV = 96.3% (94.6–97.4)	715^*^	1 ^a^	2 ^#a^	3 ^b^	1 ^a^	7	3.7% (0.7–18.3)	14.3% (2.6–51.3)
	Total	787	89	95.4% (87.3–98.4)	760	13	4	9	1	27	23.6% (15.2–33.8)	77.8% (59.2–89.3)

Abbreviation: *CI* confidence interval; *PPV* Positive predictive value; *NPV* Negative predictive value. ^*^, include 26 false negative Xpert results; ^#^, include 1 false negative Xpert result (1 true positive AFB smear result); ^a^, include 1 false positive AFB smear result; ^b^, include 3 false positive AFB smear results

Xpert MTB/RIF semi-quantitative results defined by the manufacturer as follows: positive very low (cycle threshold, CT > 28), low (22 < CT≤ 28), medium (16 < CT≤ 22) or high (CT ≤ 16)

AFB smear microscopy results were graded according to American Thoracic Society scale: Negative, 0 AFB/300 fields; Positive 1+, 1–9 AFB/100 fields; Positive 2+, 1–9 AFB/10 fields; Positive 3+, 1–9 AFB/field; Positive 4+, > 9 AFB/field

### Comparison of Xpert MTB/RIF and IGRA in AFB smear-negative patients

Among the 27 AFB positive cases, 21 confirmed TB cases (23.6%, 21/89, 19 PTB, and 2EPTB) were detected, with a PPV of 77.8%; the pooled sensitivity, specificity, PPV, and negative predictive value (NPV) of Xpert were 95.2, 100, 100, and 85.7%, respectively, all higher than IGRA (90.5, 50.0, 86.4, and 60.0%, respectively). Obviously, 68 confirmed TB (76.4%, 68/89, 33 PTB, 15 EPTB, and 20 TP) were detected using Xpert and/or IGRA in AFB smear-negative patients (*n* = 760, Table S2); the sensitivity of Xpert (61.8, 95% CI 49.9–72.4%) was significantly lower than IGRA (94.1%, 95 CI 85.8–97.7%) (*P* < 0.001), while the specificity of the former (99.6, 95% CI 98.7–99.9%) was significantly higher than the latter (57.5, 95% CI 53.8–61.2%) (*P* < 0.001). In detail, the sensitivity of Xpert was 84.9, 73.3, and 15.0% for detecting presumptive PTB, EPTB, and TP cases, respectively, which were lower than IGRA (100, 80.0, and 95.0%); while the specificity of Xpert (99.6, 99.4, and 100%) was significantly higher than IGRA (56.1, 66.9, and 45.2%) (*P* < 0.001).

## Discussion

With the progress of molecular biology technology, the use of Xpert for TB detection in China is increasing. In total, 787 patients simultaneously tested with Xpert, AFB, and IGRA at the PKUPH from November 2016 to October 2018 were enrolled, and according to ROC curves (Fig. 2**)**, Xpert showed the best diagnostic performance (AUC = 0.846, sensitivity = 69.7% and specificity = 99.6%), and significantly higher than AFB and IGRA (*P* < 0.001). The high specificity of Xpert across all specimens highlights its utility as a rule-in test for TB diagnosis, and can be used to reliably inform the start of TB treatment when positive [17].

This study confirms that Xpert showed high sensitivity (88.5%) in the diagnosis of PTB, as reported by previous studies (82–88%) [18]. It is interesting to note that lung tissue (*n* = 52), a specimen that was less evaluated in previous studies, showed the highest sensitivity (100%, 7/7) among pulmonary specimens, probably due to higher bacteria loads in diseased tissues than sputum, BALF, and FOB. Consistent with the literature (68–94%) [17], this research found that Xpert also showed high sensitivity (76.5%) in the diagnosis of EPTB. When pleural fluid was used to diagnose TP, the high specificity (100%) of Xpert suggested its high value in confirming TP diagnosis; however, the low sensitivity (15.0%), which was similar to previous studies (14–34%) [17, 19, 20], indicated that Xpert is of limited value for TP screening. Low sensitivity may be attributed to the presence of PCR inhibitors in pleural fluid [20], or the loading of MTB is too low that the detection limit cannot be reached even by centrifugation. Thus, this study together with previous literature, did not recommend the use of pleural fluid as a specimen of Xpert for the diagnosis of TP, until pleural fluid was optimized for improved sensitivity. Pleural biopsy may be a better alternative, but an invasive procedure was required, and the sensitivity (45%) improvement was still not ideal [19].

There were three Xpert false positive cases in this study, one sputum (CT = 26.8, AFB negative and IGRA positive), one BALF (CT = 29.3, AFB negative and IGRA positive), and one intestinal tissue (CT = 27.0, AFB negative and IGRA negative). According to previous studies, the false-positive results of Xpert may occur in patients with prior TB, and Xpert may detect cell-free DNA rather than DNA in cells [21].

The false positives of IGRA are common in this survey (false positives rate, i.e., FPR was 46.5%), which is consistent with previous research in China (FPR was 43.6%) [22], mainly because China is a country with high burden of TB, and the prevalence of latent TB is as high as 44.5% [23]; in addition, for patients with previous TB, there may be a long-term presence of antigen-specific memory T cells in the body, resulting in false positive results [24]. Thus, IGRA was not suggested to be used alone for the diagnosis of active PTB in high-burden TB settings. There were few false negatives of IGRA in this study (six cases): the reduced immune response or immunosuppression of T lymphocyte function against MTB-specific antigens due to increasing age, long-term hospitalization (> 6 months), overweight, obesity, concomitant immune system instability, HIV infection and other immunosuppressive diseases, and the use of steroid drugs may lead to false negative results of IGRA [24]. However, in consideration of sampling for the detection of EPTB and TP, which is still challenging in peripheral level laboratories in China, the highly sensitive IGRA was recommend for use as a supplementary test for TP and EPTB diagnosis.

The specificity of AFB was high (99.1%), and false positive cases (six cases) may mainly be due to infections caused by nontuberculous mycobacteria. Low sensitivity of AFB (23.6%; 68 AFB false negative cases occurred) due to its high detection limit (5000 ~ 10,000 CFU/mL, while the detection limit of Xpert was 131 CFU/mL in sputum) [25, 26]. Thus, its utility as a rule-in test for TB diagnosis was limited, and it is not recommended for use alone for early TB diagnosis. Notably, for smear-positive cases, Xpert (95.2%) had higher sensitivity than IGRA (90.5%).

The positive rate of rifampicin-resistant TB in this study was 12.9% (8/62) (for reference only, as there is no gold standard for drug-resistant TB confirmation in this study), higher than the 5th Chinese TB survey in 2010 (6.8%) [27], but lower than the previous research in a tertiary TB referral hospital in Beijing, China (16.9% in 2006 and 30.5% in 2012) [28], which may be due to the higher proportion of previously-treated TB cases in the latter study, as previous treatment is a well-known risk factor for drug-resistant TB. Considering that the importance of early and effective treatment has been highlighted, it is necessary to monitor the occurrence of TB and rifampicin resistance rate, and maintain good practice in TB prevention, care, and treatment in non-TB specialized hospitals.

This study has some limitations. First, this was a single-center retrospective study, thus the overall relevant scope of our findings was limited. Second, PKUPH is a non-TB specialized comprehensive teaching hospital, and TB culture was not carried out in many patients, thus the culture method was not analyzed and failure to compare with CRS (data is shown in Table S3); besides, the culture-based MTB antimicrobial susceptibility test was not conducted, thus rifampicin-resistant TB was not verified. Third, the low priori power (< 0.8) (power analyses was done by NCCS-PASS 11 program) for assessment of the partial result of Xpert, AFB smear microscopy, and IGRA in detecting presumptive TB, as well as different specimens tested using Xpert may cause type of error and overlook significant differences due to the small sample size. However, the sensitivity, specificity, PPV, NPV, and corresponding 95% CI of the different methods used in our study will be helpful in designing future multicenter prospective studies covering different regions of China.

## Conclusions

The traditional AFB smear microscopy method showed low sensitivity and high specificity, thus it is not recommended for use alone for early TB diagnosis. Though the specificity of IGRA is relatively low, it still has a certain diagnostic value, especially when assisting diagnosis of EPTB, where the specimens are difficult to sample and TP, where the performance of Xpert is relatively poor. Xpert showed both high sensitivity and high specificity, even in AFB smear-negative patients; however, pleural fluid is not recommended as a specimen for Xpert for the diagnosis of TP. The simultaneous use of these three methods could help screening 96.6% of TB patients, but it should be noted that the PPV (7.3%) is poor when only IGRA is positive. In future studies, the treatment process of pleural fluid should be optimized, and pleural biopsy may be sent together or as a substitute to improve Xpert detection efficiency.

## Supplementary information

**Additional file 1:** Supplementary material 1. Sample processing for Xpert MTB/RIF assay.

**Additional file 2: Table S1.** Age and gender information of the patients in this study.

**Additional file 3: Table S2.** Performance of Xpert MTB/RIF and IGRA in AFB smear negative cases.

**Additional file 4: Table S3.** A comparison of composite reference standard (CRS) and TB culture.

**Additional file 5: Figure S1.** Correspondence between smear grades and cycle threshold (CT) values of Xpert MTB/RIF.

## Data Availability

The datasets used and analyzed during the current study are available from the corresponding author upon reasonable request.

## Abbreviations

AFB: Acid-fast bacilli
AUC: Area under the curve
BALF: Bronchoalveolar lavage fluid
CI: Confidence interval
CLSI: Clinical and Laboratory Standards Institute
CRS: Composite reference standard
CSF: Cerebrospinal fluid
CT: Cycle threshold
EPTB: Extrapulmonary tuberculosis
FOB: Fiberoptic bronchoscopy
IGRA: Interferon-gamma (IFN-γ) release assay
MTB: *Mycobacterium tuberculosis*
NPV: Negative predictive value
PKUPH: Peking University People’s Hospital
PPV: Positive predictive value
PTB: Pulmonary tuberculosis
ROC: Received operating characteristic
TB: Tuberculosis
TP: Tuberculous pleurisy
WHO: World Health Organization
Xpert: Xpert MTB/RIF
