# Supplementary material for: A retrospective study on Xpert MTB/RIF for detection of tuberculosis in a teaching hospital in China
Source: BMC Infect Dis. 2020 May 24;20:362. doi: 10.1186/s12879-020-05004-8 (PMC7245878; doi:10.1186/s12879-020-05004-8)
Supplement: Supplementary file 1 — Additional file 1: Supplementary material 1. Sample processing for Xpert MTB/RIF assay. [file 12879_2020_5004_MOESM1_ESM.docx]

**Supplementary material 1**

Sample processing for Xpert MTB/RIF assay

The “purulent samples”, e.g. sputum, purulent fiberoptic bronchoscopy, and purulent pleural fluid, were digested with equal volume of 4% NaOH for 10 minutes, and 1 ml of digested sample was mixed with 1:2 ratio using sample reagent (SR) of the kit for 10 minutes at room temperature. The specimen was then vortexed for 10 seconds and left to incubate for an additional 5 minutes. Then 2 ml of diluted samples were transferred into single-use disposable cartridge of Xpert.

The “solid samples”, e.g. tissue and biopsy, were ground with equal volume of sterile water, and 1 ml tissue homogenate was mixed with 1:2 ratio using SR for 10 minutes at room temperature. The specimen was then vortexed for 10 seconds and left to incubate for an additional 5 minutes. Then 2 ml of diluted samples were transferred into single-use disposable cartridge of Xpert.

The “fluid sample”, e.g. urine, CSF, and clear pleural fluid, were centrifuged at 10000 g for 10 min, and the centrifugal sediment was mixed with 2 ml SR for 10 minutes at room temperature; the specimen was then vortexed for 10 seconds and left to incubate for an additional 5 minutes. Then 2 ml of diluted samples were transferred into single-use disposable cartridge of Xpert.

**References:**

1. Bahr et al., Diagnostic accuracy of Xpert MTB/RIF Ultra for tuberculous meningitis in HIV-infected adults: a prospective cohort study. Lancet Infect Dis. 2018; 18(1):68-75.
2. Xie et al., Evaluation of a Rapid Molecular Drug-Susceptibility Test for Tuberculosis. N Engl J Med. 2017; 377(11):1043-1054.
3. Siddiqi et al., A Prospective Cohort Study on the Performance of CSF Xpert MTB/RIF, CSF and Urine LAM Lateral Flow Assay for the Diagnosis of Tuberculous Meningitis in Zambia. J Clin Microbiol. 2019; 57(8). pii: e00652-19.
