# Supplementary material for: A retrospective study on Xpert MTB/RIF for detection of tuberculosis in a teaching hospital in China
Source: BMC Infect Dis. 2020 May 24;20:362. doi: 10.1186/s12879-020-05004-8 (PMC7245878; doi:10.1186/s12879-020-05004-8)
Supplement: Supplementary file 2 — Additional file 2: Table S1. Age and gender information of the patients in this study. [file 12879_2020_5004_MOESM2_ESM.docx]

**Table S1** Age and gender information of the patients in this study

| Characteristic | All (N =787) | Conﬁrmed TB  (n = 89) | Non TB  (n = 698) | *P* value |
| --- | --- | --- | --- | --- |
| **Age** | | | | |
| Average age (±SD^*^) | 55.4 (±18.5) | 53.1 (±20.2) | 55.7 (±18.3) | 0.252^a^ |
| Children (≤14 years) | 5 (0.6%) | 0 (0%) | 5 (100%) | 0.876, 1.000^b^ |
| Adult (15-64 years) | 508 (64.5%) | 63 (12.4%) | 445 (87.6%) | 0.876, 0.221 ^c^ |
| Elder (>65 years) | 274 (34.9%) | 26 (9.5%) | 248 (90.5%) | 1.000, 0.221 ^d^ |
| **Gender** | | | | |
| Male | 434 (55.1) | 46 (10.6%) | 388 (89.4%) | 0.486^e^ |
| Female | 353 (44.9) | 43 (12.2%) | 310 (87.8%) |  |

*, Standard Deviation

Statistical analysis was done with IBM SPSS Statistics 20:

^a^, Kruskal-Wallis test;

^b^, Pearson's chi-squared test between Children & Adult, and between Children & Elder;

^c^, Pearson's chi-squared test between Adult & Children, and between Adult & Elder;

^d^, Pearson's chi-squared test between Elder & children, and between Elder & Adult;

^e^, Pearson's chi-squared test.
