# Supplementary material for: A retrospective study on Xpert MTB/RIF for detection of tuberculosis in a teaching hospital in China
Source: BMC Infect Dis. 2020 May 24;20:362. doi: 10.1186/s12879-020-05004-8 (PMC7245878; doi:10.1186/s12879-020-05004-8)
Supplement: Supplementary file 3 — Additional file 3: Table S2. Performance of Xpert MTB/RIF and IGRA in AFB smear negative cases. [file 12879_2020_5004_MOESM3_ESM.docx]

**Table S2** Performance of Xpert MTB/RIF and IGRA in AFB smear negative cases

|  | Sensitivity (95% CI) | Specificity (95% CI) | Positive predictive value (95% CI) | Negative predictive value (95% CI) |
| --- | --- | --- | --- | --- |
| **Suspected PTB cases (n=509)** |  |  |  |  |
| Xpert MTB/RIF | 84.9% (69.1-93.4) | 99.6% (98.5-99.9) | 93.3% (78.7-98.2) | 99.0% (97.6-99.6) |
| IGRA | 100% (89.6-100.0) | 56.1% (51.6-60.5)^*^ | 13.6% (9.9-18.5) | 100% (98.6-100) |
| **Suspected EPTB cases (n=169)** |  |  |  |  |
| Xpert MTB/RIF | 73.3% (48.1-89.1) | 99.4% (96.4-99.9) | 91.7% (64.6-98.5) | 97.5% (93.6-99.0) |
| IGRA | 80.0% (54.8-93.0) | 66.9% (59.1-73.8)^*^ | 19.1% (11.3-30.4) | 97.2% (92.0-99.0) |
| **Suspected TP cases (n=82)** |  |  |  |  |
| Xpert MTB/RIF | 15.0% (5.2-36.0) | 100% (94.2-100) | 100% (43.9-100) | 78.5% (68.2-86.1) |
| IGRA | 95.0% (76.4-99.1)^*^ | 45.2% (33.4-57.5)^*^ | 35.9% (24.3-49.3) | 96.6% (82.8-99.4) |
| **Total (n=760)** |  |  |  |  |
| Xpert MTB/RIF | 61.8% (49.9-72.4) | 99.6% (98.7-99.9) | 93.3% (82.1-97.7) | 96.4% (94.7-97.5) |
| IGRA | 94.1% (85.8-97.7)^*^ | 57.5% (53.8-61.2)^*^ | 17.9% (14.3-22.2) | 99.0% (97.5-99.6) |

Abbreviation: AFB, acid-fast bacilli; IGRA, interferon-gamma release assay; PTB, pulmonary tuberculosis; EPTB, extra-pulmonary tuberculosis; TP, tuberculous pleurisy; CI, confidence interval.

^*^, statistical differences between the sensitivity or specificity of IGRA and Xpert MTB/RIF, chi-square (χ2) test, *P*<0.001.
