# Supplementary material for: A retrospective study on Xpert MTB/RIF for detection of tuberculosis in a teaching hospital in China
Source: BMC Infect Dis. 2020 May 24;20:362. doi: 10.1186/s12879-020-05004-8 (PMC7245878; doi:10.1186/s12879-020-05004-8)
Supplement: Supplementary file 4 — Additional file 4: Table S3. A comparison of composite reference standard (CRS) and TB culture. [file 12879_2020_5004_MOESM4_ESM.docx]

**Table S3 A comparison of composite reference standard (CRS) and TB culture**

| Specimen type | No. (%) | Culture | CRS | |
| --- | --- | --- | --- | --- |
|  |  |  | Positive | Negative |
| Bronchoalveolar lavage fluid | 204 (25.9%) | Positive | 0 | 0 |
|  |  | Negative | 0 | 12 |
|  |  | No culture result | 7 | 185 |
| Sputum | 165 (21.0%) | Positive | 0 | 0 |
|  |  | Negative | 3 | 10 |
|  |  | No culture result | 23 | 129 |
| Fiberoptic bronchoscopy | 105 (13.3%) | Positive | 0 | 0 |
|  |  | Negative | 1 | 9 |
|  |  | No culture result | 10 | 85 |
| Pulmonary tissue | 52 (6.6%) | Positive | 1 | 0 |
|  |  | Negative | 4 | 11 |
|  |  | No culture result | 2 | 34 |
| Other pulmonary specimen | 7 (0.9%) | Positive | 0 | 0 |
|  |  | Negative | 0 | 0 |
|  |  | No culture result | 1 | 6 |
| Intestine tissue | 53 (6.7%) | Positive | 0 | 0 |
|  |  | Negative | 0 | 0 |
|  |  | No culture result | 0 | 53 |
| Cerebrospinal fluid | 42 (5.3%) | Positive | 0 | 0 |
|  |  | Negative | 0 | 3 |
|  |  | No culture result | 2 | 37 |
| Ascitic fluid | 18 (2.3%) | Positive | 0 | 0 |
|  |  | Negative | 0 | 2 |
|  |  | No culture result | 0 | 16 |
| Joint cavity fluid | 13 (1.7%) | Positive | 1 | 0 |
|  |  | Negative | 0 | 5 |
|  |  | No culture result | 1 | 6 |
| lymph node | 12 (1.5%) | Positive | 0 | 0 |
|  |  | Negative | 0 | 1 |
|  |  | No culture result | 5 | 6 |
| Urine | 11 (1.4%) | Positive | 0 | 0 |
|  |  | Negative | 0 | 1 |
|  |  | No culture result | 3 | 7 |
| Other extrapulmonary specimen | 23 (1.7%) | Positive | 2 | 0 |
|  |  | Negative | 1 | 7 |
|  |  | No culture result | 2 | 11 |
| Pleural fluid | 82 (10.4%) | Positive | 0 | 0 |
|  |  | Negative | 1 | 5 |
|  |  | No culture result | 19 | 57 |
| Total | 787 | Positive | 4 | 0 |
|  |  | Negative | 10 | 66 |
|  |  | No culture result | 75 | 632 |

*****Bronchoalveolar lavage fluid, sputum, and fiberoptic bronchoscopy were cultured in Löwenstein–Jensen (LJ) solid culture (Baso, China); while pulmonary tissue and other specimens were cultured in both solid culture with LJ and liquid culture with “BACTEC Myco/F LYTIC Culture Vial” (Becton, Dickinson and Company, US).
