# Supplementary figures and images for: A retrospective study on Xpert MTB/RIF for detection of tuberculosis in a teaching hospital in China
Source: BMC Infect Dis. 2020 May 24;20:362. doi: 10.1186/s12879-020-05004-8 (PMC7245878; doi:10.1186/s12879-020-05004-8)

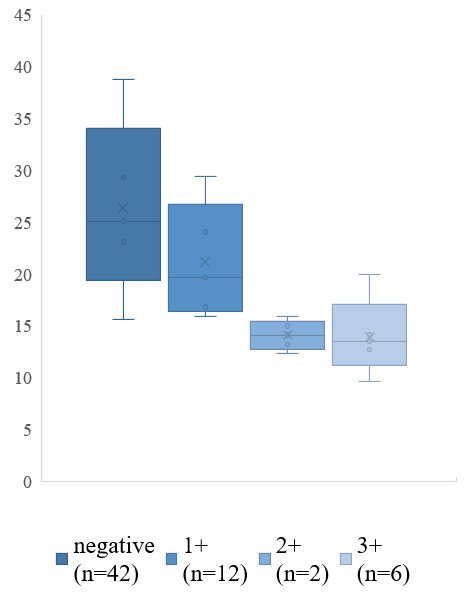

Supplement: Supplementary file 5 — Additional file 5: Figure S1. Correspondence between smear grades and cycle threshold (CT) values of Xpert MTB/RIF. [file 12879_2020_5004_MOESM5_ESM.png]
